# Relapses

**Published:** 1858-05

**Authors:** 


### RELAPSES.

"FALLING back"—that is the meaning of the term in medicine, where a person is getting well, and by some untoward event his convalescence is arrested: Nature has not power to recuperate again, and death is the very general result. A person recovering from a serious illness is like one coming to life again, and should be watched over with the tenderest care. Within a year, a very estimable lady, KATE H., was slowly recovering from an attack of typhoid fever. Every day the sunshine of hope was clearer and more genial, and those who loved her most were looking joyfully to a future, all the happier from the narrow escape. As is usual in such cases, the appetite grew faster than the ability to digest the food it demanded; besides, it was capricious, and called for unusual things. In this instance, there was a strong desire for a sweet potato. Her husband, with commendable prudence, consulted the physician, who had dismissed the case, as not needing further special attendance. Unfortunately, permission was given to take a small one. It rested like a heavy weight in the stomach; Nature could not throw it off; and in a few days the patient passed away, leaving her first-born, of a few months old, a motherless child.

Within a day or two, one of our citizens of distinction died under precisely similar circumstances; and let the lesson be deeply impressed on the mind of every reader, that when a a person *is getting well* of any serious disease, the utmost precaution is imperative, especially in the avoidance of over-eating and over-exercise—very great moderation should be steadily observed in both.

From this medical fact, our religious readers may learn a lesson of incalculable value, in connection with the events of the last few months, which have so fully engaged their attention; and it is this: that in order to make the effects of the "Great Awakening" *permanently* beneficial to our city, and State, and common country, no more assiduous, and delicate, and considerate care, does the feeble convalescent from a terribly fatal disease require, than does that class of persons now designated by "New Converts," who, like the tiny, brittle plants of spring, are just emerging into a new life, which the slightest touch of rudeness would snap off, and which are so sedulously fenced around with little sticks, to protect them from harm, until they are more able to stand alone. And not a whit wiser is it to say that the seeds were not good, because the stem which sprang therefrom could stand no rude shock, than to say a "revival" is not a reality, because the subjects of it, left alone too soon, are broken off in the bud by uncouth hands, by strong temptations, and by wily snares.

A plant, just peering above the ground, needs more care than when it was only a seed. A child or man just converted, needs a tenderer care than before, needs the daily watchfulness, and counsel, and sympathy, and encouragement of all who truly love them. These things being so, let every good man and woman feel as if they were personally responsible for preventing the "Great Awakening" from becoming a hissing and a by-word a few short years hence; let every new convert known to them be considered in the light of a "god-child," in the light of the frailest and tiniest plant, which the most incessant and most delicate attention will cause to blow into a magnificent flower, which shall charm every beholder. It is from neglect in these points, the almost entire failure of care, as soon as a person becomes a member of the Church, that very much of the prejudice in the minds of many good people has grown up in reference to "Revivals." As well may the skill of the good physician be called in question, after he has actually saved his patient's life, because the nurse failed to bestow the proper care. Relapses in disease are as disastrous as those in religion, and are always to be deplored.

